# Finishing Plate-Work

**Published:** 1860-01

**Authors:** J. L. Suesserott


### FINISHING PLATE-WORK.

BY J. L. SUESSEROTT.

HAVING a consciousness that your Journal, and the one which formerly occupied its place in our literature, has given me many valuable hints—in value more than ten times the amount of subscription price—with your permission, I will give to the profession my method of finishing plates; a method which may possibly be familiar to many of my professional brethren, but one which I am fully persuaded is not generally adopted.

The first step is to procure, and attach to the lathe, a three or four-pronged fork, or a screw such as is used for withdrawing a load from a gun. Upon this a good, smooth cork is fixed, and, with a sharp knife, turned to any desired shape. The cork is then saturated with water as well as it can be, and powdered pumice placed upon it. If we have been careful to remove all excess of solder from our work—which can easily be done by a bur attached to the lathe—we can, with the cork and pumice, make a very smoothe surface, and this can be still more perfectly accomplished by substituting a very finely powdered spar for the pumice, after we have removed the larger scratches with the latter. By continuing the cork for a little while after the above-named powders have been used off, we avoid the use of the Scotch-stone; and finally we dispense with the burnisher, by taking a new cork with a piece of chamois or buckskin stretched upon it, and going over the plate in the same manner as before, with the lathe revolving very rapidly.

A higher color can be given to the plate, by the use of the burnisher after the above proceeding, but we can certainly not produce a smoother surface.

Some precaution is necessary by those who have never used the lathe in finishing plates; in the first place, a careless use of the bur, in removing the excess of solder, might result in the weakening of the piece by removing more than necessary, or, what would be still worse, holes might be eat entirely through the plate. Again, in polishing, if a little care is not taken, the fork or screw, whichever is used, may pass through the cork, and, before the operator is aware, he will have inflicted an injury that will be difficult to repair. A small amount of experience—that which is essential in the proper performance of every nice operation—will enable almost any one, even those, to use a common expression, "whose fingers are all thumbs," to finish their work in about one-eighth of the time that the most expert workman would require for the accomplishment of the same by the old method.

As this is the age of locomotives and telegraphs, I have been induced to think that anything that would save time would be gladly received.—*Dental Cosmos.*

